# Warming and CO_2_ Enhance Arctic Heterotrophic Microbial Activity

**DOI:** 10.3389/fmicb.2019.00494

**Published:** 2019-03-20

**Authors:** Dolors Vaqué, Elena Lara, Jesús M. Arrieta, Johnna Holding, Elisabet L. Sà, Iris E. Hendriks, Alexandra Coello-Camba, Marta Alvarez, Susana Agustí, Paul F. Wassmann, Carlos M. Duarte

**Affiliations:** ^1^Departament de Biologia Marina i Oceanografia, Institut de Ciències del Mar (CSIC), Barcelona, Spain; ^2^Centro Oceanográfico de Canarias (IEO), Santa Cruz de Tenerife, Spain; ^3^Global Change Research Group, IMEDEA (CSIC-UIB) Institut Mediterrani d’Estudis Avançats, Esporles, Spain; ^4^Arctic Research Centre, Aarhus University, Aarhus, Denmark; ^5^Red Sea Research Center, King Abdullah University of Science and Technology, Thuwal, Saudi Arabia; ^6^Instituto Español de Oceanografía, A Coruña, Spain; ^7^Department of Arctic and Marine Biology, Faculty of Biosciences, Fisheries and Economics, University of Tromsø, Tromsø, Norway

**Keywords:** *p*CO_2_, temperature, microbial food-webs, viral life cycle, Arctic Ocean

## Abstract

Ocean acidification and warming are two main consequences of climate change that can directly affect biological and ecosystem processes in marine habitats. The Arctic Ocean is the region of the world experiencing climate change at the steepest rate compared with other latitudes. Since marine planktonic microorganisms play a key role in the biogeochemical cycles in the ocean it is crucial to simultaneously evaluate the effect of warming and increasing CO_2_ on marine microbial communities. In 20 L experimental microcosms filled with water from a high-Arctic fjord (Svalbard), we examined changes in phototrophic and heterotrophic microbial abundances and processes [bacterial production (BP) and mortality], and viral activity (lytic and lysogenic) in relation to warming and elevated CO_2_. The summer microbial plankton community living at 1.4°C *in situ* temperature, was exposed to increased CO_2_ concentrations (135–2,318 μatm) in three controlled temperature treatments (1, 6, and 10°C) at the UNIS installations in Longyearbyen (Svalbard), in summer 2010. Results showed that chlorophyll *a* concentration decreased at increasing temperatures, while BP significantly increased with *p*CO_2_ at 6 and 10°C. Lytic viral production was not affected by changes in *p*CO_2_ and temperature, while lysogeny increased significantly at increasing levels of *p*CO_2_, especially at 10°C (*R*^2^ = 0.858, *p* = 0.02). Moreover, protistan grazing rates showed a positive interaction between *p*CO_2_ and temperature. The averaged percentage of bacteria grazed per day was higher (19.56 ± 2.77% d^-1^) than the averaged percentage of lysed bacteria by virus (7.18 ± 1.50% d^-1^) for all treatments. Furthermore, the relationship among microbial abundances and processes showed that BP was significantly related to phototrophic pico/nanoflagellate abundance in the 1°C and the 6°C treatments, and BP triggered viral activity, mainly lysogeny at 6 and 10°C, while bacterial mortality rates was significantly related to bacterial abundances at 6°C. Consequently, our experimental results suggested that future increases in water temperature and *p*CO_2_ in Arctic waters will produce a decrease of phytoplankton biomass, enhancement of BP and changes in the carbon fluxes within the microbial food web. All these heterotrophic processes will contribute to weakening the CO_2_ sink capacity of the Arctic plankton community.

## Introduction

The Arctic Ocean is warming at two to three times the global rate and is experiencing accelerated ice loss with a historical minimum reached in the summer of 2012 (Parkinson and Comiso, 2013). Furthermore, it is predicted to be free of ice during the summer as early as 2050 (Intergovernmental Panel on Climate Change [IPCC], 2014). Due to the higher solubility of CO_2_ in cold waters, polar regions are particularly vulnerable to ocean acidification by anthropogenic CO_2_ (Shadwick et al., 2013). The Arctic Ocean is characterized by very low concentrations of *p*CO_2_ < 200 μatm in the spring and early summer due to high net primary productivity (Takahashi et al., 2002; Vaquer-Sunyer et al., 2013). However, increased atmospheric *p*CO_2_ is expected to reach values ≥1,000 μatm by 2,100 (Meehl et al., 2007; United States Environmental Protection Agency [USEPA], 2013), while enhanced air-sea fluxes with decreasing ice cover (Bates et al., 2006) will result in increased *p*CO_2_ uptake in Arctic water, with thresholds for carbonate saturation states predicted to be reached as early as 2020 (Popova et al., 2014).

Previous experimental studies have shown, that warming (beyond a threshold of 5°C) triggers a decrease of phytoplankton biomass and net primary production in the Arctic (Holding et al., 2013; Coello-Camba et al., 2015). This will favor an increase of bacterial growth which is translated in excess community respiration over gross primary production (Holding et al., 2013). Then, a major effect of warming produce changes in the plankton carbon flow pattern, enhancing bacterial processing of DOC (and resulting increased CO_2_ production). This has been confirmed by many other experimental studies (e.g., Wohlers et al., 2009). In addition, experimental and *in situ* results point out that CO_2_ increase may fertilize planktonic primary producers in the European Arctic Ocean (Engel et al., 2013), however, the effect is temperature dependent (Beardall et al., 2009). Recently, Coello-Camba et al. (2014), Holding et al. (2015) and Sanz-Martin et al. (2018) showed that CO_2_ has a fertilizing effect on primary production at lower temperatures but that effect become negligible at increasing temperatures. These drivers act in opposite directions, with CO_2_ enhancing primary production while temperature affecting primary production negatively (Keys et al., 2018). A positive effect of increasing temperature and *p*CO_2_ on marine bacterial communities is also observed, favoring their growth as a consequence of selecting different more active phylotypes (e.g., Lindh et al., 2013; Piontek et al., 2013).

Furthermore, it has also been assessed that the increases of bacterial production (BP) due to warming, triggers greater bacterial carbon transfer to higher trophic levels rather than the flux of dissolved organic carbon from bacteria lysed by viruses (Lara et al., 2013; Maranger et al., 2015). This could be due to the observed success of lysogeny respect to lysis in warming conditions (Lara et al., 2013). Nevertheless, there is still limited information available on the interacting effect of increasing CO_2_ concentrations and temperature on mortality (grazing due to protists vs. rate of lysed bacteria by viruses) and even less knowledge of its effects on the viral life style (lytic versus lysogeny). Based in the above information in our study, we will expect after applying these two stressors on the whole microbial planktonic community, an increase of BP mainly at higher temperatures, followed by an increase of grazing rates by protists. Bacterial mortality rates due to viruses, will depend on lytic -lysogenic infection cycle, where according to Jiang and Paul (1994), lysogenic infection should prevail over lytic production in oligotrophic conditions with low BP, while lytic infection should increase when BP is high.

Here, we examined the responses of phytoplankton biomass and heterotrophic microbial communities, including viruses, to the concurrent changes in warming and CO_2_ increase, attempting to discriminate whether they may have a direct or synergistic effect. To unravel the interactive effect of both temperature and CO_2_, we used experimental 20 L microcosms to assess the changes of phytoplankton and heterotrophic microbial abundances and processes (BP and mortality) in water from a high-Arctic fjord (Svalbard). We continuously bubbled CO_2_ (ranging from 135 to 2318 μatm) in three controlled temperature treatments (1, 6, and 10 °C). The chosen range for temperature covered the projected increases plus 2–3°C expected in the Arctic fort the coming century (ACIA, 2004). For CO_2_ the low range was chosen much lower than present day CO_2_ concentrations, as *p*CO_2_ in Arctic waters is often undersaturated compared to *p*CO_2_ in the atmosphere, and it is not uncommon to find values of 150 ppm. The high range was not necessarily selected, but just consequence of controlled CO_2_ increase plus respiration inside the mesocosms resulting in a high range. While it is far from expected concentrations by the end of the century (United States Environmental Protection Agency [USEPA], 2013) it is also an experimental extreme case scenario, which is interesting to determine overall trends.

Then, under these experimental conditions we evaluated the relationship between phytoplankton biomass (i.e., chlorophyll *a* concentration, and phototrophic pico/nanoflagellates abundance) and BP, and its fate (i.e., grazing on bacteria by protists, rates of bacterial lysis) as well as viral infection type (lytic vs. lysogeny). Finally, in order to confirm the consistency of our experimental results we compared the BP obtained at increasing *p*CO_2_ and temperature with available field data (from the Greenland Sea and North of Svalbard; Boras et al., 2010) and *p*CO_2_ data from Alvarez (unpublished) during the ATOS-I cruise.

## Materials and Methods

### Experimental Design and Set Up

Seawater for the experiment was collected on June 23, 2010 just outside of Isfjorden Svalbard (78.0826° N, 13.4734° E) aboard R/V Viking Explorer. Seawater was pumped on board from a depth of 1–2 m (salinity: 33.50; temperature: 1.41°C), filtered through 200 μm in order to avoid large zooplankton and distributed in 60 L carboys previously treated with 0.1 N HCl for at least 48 h and thoroughly rinsed with seawater from the same sampling site. Seawater from different carboys (60 L) was pooled together in larger tanks (280 L) and transferred to acid-washed 20 L clear polycarbonate Nalgene^TM^ bottles, which served as the experimental microcosms. The experiment was conducted in a temperature regulated cold room (set at 4.5 ± 1°C) at the University Center in Svalbard (UNIS), Longyearbyen. The experimental design consisted of six replicated 20 L microcosms of three levels of temperature treatments (1, 6, and 10°C), a total of 18 microcosms, into which we constantly bubbled CO_2_ gas (Supplementary Figure S1). The eighteen 20 L bottles were submerged in nine 280 L tanks where experimental temperature was regulated by a temperature control unit (PolyScience 9600 series, precision 0.1°C) and held stable over the 13 experiment days (1°C: 1.8 ± 0.4°C; 6°C: 6.7 ± 0.7°C; 10°C: 10.3 ± 0.5°C). Afterward, the 6 microcosms replicates for each temperature treatment were divided in half, receiving bubbled air from two sets of mass flow controllers (Aalborg GFC17), (Supplementary Figure S1), which regulated a mixing ratio of gasses (CO_2_ + air mixture). Each of the two mass flow controllers was split into 3 tubes for each temperature treatment, and each of which was split again into 3 to accommodate each microcosm replicate in each temperature treatment. This allowed for 2 groups of three true replicates in each temperature treatment (Supplementary Figure S1). In each of the two groups of triplicates for each temperature treatment we tried to establish “Low” and “High” CO_2_ treatments. However, the mean (±SE) *p*CO_2_ along the duration of the experiment in the “Low” CO_2_ treatment was: 358.9 ± 38.9 μatm at 1°C, 640.5 ± 60.2 μatm at 6°C, and 559.4 ± 69.1 μatm at 10°C; whereas in the “High” CO_2_ treatment, was: 1127.5 ± 93.4 μatm at 1°C, 986.4 ± 228.5 μatm at 6°C, and 1014 ± 217.7 μatm at 10°C. Due to this high variability in *p*CO_2_ for “Low” and “High” conditions, *p*CO_2_ was treated as a continuous variable with a gradient-basis design (Holding et al., 2015), ranging from 135 to 2318 μatm, and temperature as a fixed variable. This allowed to perform analyses of covariance to determine interactions between CO_2_ and temperature.

Ambient air was collected via aquarium pumps, stripped of CO_2_ by passing through soda lime columns, and mixed with CO_2_ gas before it was delivered to the microcosms via impermeable Tygon tubing. Microcosms were aerated with air-CO_2_ mixture continuously via 5 mm diameter PTFE tubing. Airflow was adjusted using Hoffman clamps so as to avoid introducing turbulence to the microcosms; bubble size was manually controlled to be no larger than approximately 7 mm diameter (for more details see Holding et al., 2015). All plasticware used for bubbling directly into the microcosms was previously cleaned with HCl and thoroughly rinsed with seawater. Finally, the set up was completed with two fluorescent light tubes per tank (200 μmol photons s^-1^ m^-2^ determined using a 4π LI-193 LI-COR radiation sensor) providing a continuous 24 h light environment, thereby simulating the Arctic summer natural conditions.

### Carbonate Parameters

Total hydrogen ion (pH_T_) concentration of the seawater was measured spectrophotometrically using the indicator dye m-cresol purple (Sigma-Aldrich), according SOP6b, while total alkalinity (TA) was determined using open-cell titration following the SOP3b from Dickson et al. (2007). TA and pH_T_ measured at standard temperature and pressure, along with nutrient concentrations were used as input in the CO2SYS (Pierrot et al., 2006) program to calculate output carbon parameters (pH_T_, *p*CO_2_ in μatm, and tCO_2_ μmol kg^-1^), which were standardized to the average tank temperatures measured each day (for more details, see Holding et al., 2015).

### *In situ p*CO_2_

During the ATOS-I cruise, *p*CO_2_ was determined using a high-precision (±1 μatm) non-dispersive infrared gas analyzer (EGM-4, PP-systems), averaging measurements at 1 min recording interval. The closed gas stream flowing through the gas analyzer was previously equilibrated with the sampled surface seawater using a gas exchange column (MiniModule 1.25 × 9 Membrane Contactor, Celgard). Temperature was measured in the continuous system just before the EGM-4 and in the seawater inlet and corrected accordingly. Before entering the gas analyzer, the gas stream was circulated through a Calcium Sulfate column to avoid interferences from water vapor. The gas analyzer was calibrated, in this cruise, using two dry standards: pure nitrogen (0.0 μatm CO_2_) and a gas mixture of CO_2_ and N_2_ containing a CO_2_ molar fraction of 541 μatm, from Carburos Metalicos (Barcelona, Spain), which revealed an accuracy of ±2 μatm in the determinations of *p*CO_2_.

### Chlorophyll *a* Concentration, Microbial Abundances

From each microcosm, subsamples for chlorophyll *a* (Chla) concentration were collected daily. A volume of 50 ml was taken for each microcosm, subsequently filtered through Whatman GF/F filters and extracted using 90% acetone for 24 h, after which the concentration was measured fluorometrically following Parsons et al. (1984).

Subsamples of 2 ml were collected daily for viral abundance from each microcosm, fixed with glutaraldehyde (0.5% final concentration), refrigerated, quick frozen in liquid nitrogen and stored at -80°C as described in Marie et al. (1999) and Brussaard (2004). Counts were made using a FACSCalibur flow cytometer (Becton and Dickinson) with a blue laser emitting at 488 nm. Samples were stained with SYBR Green I and run at a flow rate ranging from 0.061 to 0.077 ml min^-1^. Samples of 150 ml for bacteria and protists abundance were collected from each microcosm at 0, 2, 4, 5, 8, 11, and 13 days for bacteria, and at 0, 5, 8, 11, and 13 days for pico/nanoflagellates. Subsamples of 10 and 20 ml for bacteria and pico/nanoflagellate (phototrophic and heterotrophic) abundance, respectively, were fixed with glutaraldehyde (1% final concentration), filtered through 0.2 and 0.6 μm black polycarbonate filters, respectively, and stained with DAPI (4,6-diamidino-2-phenylindole) (Porter and Feig, 1980) to a final concentration of 5 μg mL^-1^ (Sieracki et al., 1985), and counted by epifluorescence microscopy (Olympus BX40-102/E, at 1,000×). Pico and nanoflagellates showing red-orange fluorescence and/or plastidic structures in blue light (B2 filter) were considered phototrophic forms (PF), while colorless flagellates showing yellow fluorescence were counted as heterotrophic pico/nanoflagellates (HF). Finally, 100 ml samples were fixed with acidic lugol (2% final concentration) to estimate ciliate abundance and the abundance of the phagotrophic dinoflagellate *Gyrodinium* sp. Aliquots of the fixed samples (50-100 ml) were sedimented for 24–48 h before enumeration. Both the ciliates and *Gyrodinium* sp. were counted in an inverted microscope (Zeiss AXIOVERT35, at 400×).

### Bacterial Production

Bacterial production in the experiment as well as *in situ* (ATOS-I cruise) was measured by incorporation of radioactive ^3^H-leucine (Kirchman et al., 1985); modified by Smith and Azam (1992). Aliquots of 1.5 ml were taken at the beginning and after 2, 5, 8, 11, and 13 days from each microcosm and were dispensed into four 2-ml vials plus two TCA-killed control vials. Next, 48 μl of a 1 μM solution of ^3^H-leucine was added to the vials providing a final concentration of 40 nM. Incubations were run for 2–3 h in the same thermostatic chambers as the experimental microcosms and terminated with TCA (50% final concentration). Then, tubes were centrifuged for 10 min at 12000 rpm. Pellets were rinsed with 1.5 ml of 5% TCA, stirred and centrifuged again. Supernatant was removed and 0.5 ml of scintillation cocktail was added. The vials were counted in a Beckman scintillation counter. For each time point, BP was calculated according to the equation:

BP = Leu × CF [μg C L^-1^ d^-1^], where Leu is the ^3^H-leucine incorporation (pmol L^-1^ d^-1^), and CF is the conversion factor (1.5 kg C mol Leu^-1^) (Kirchman, 1993).

### Grazing Rates, Viral Production and Rates of Lysed Bacteria

Measurements of lytic viral production (LVP), lysogenic viral production (LysoVP), bacterial mortality due to protists (grazing rates, GZ) and viruses (bacteria lysed by viruses, BLV) were done at the beginning (only for GZ) and at 5, 8, and 13 days of the experiment for all treatments. Bacterial mortality due to protists was evaluated following the fluorescent-labeled bacteria (FLB) disappearance method (Sherr et al., 1987; Vázquez-Domínguez et al., 1999). Eighteen 1.5-L sterile bottles were filled with 0.5-L aliquots of seawater from each of the experimental microcosms. For each temperature treatment, we added an extra bottle filled with 0.5 L, 0.2 μm filtered water (grazer-free water) as a control. Then for each temperature treatment we obtained two group of triplicates, where each microcosm was bubbled with CO_2_ from the corresponding mass flow system (Supplementary Figure S1). Each bottle was inoculated with FLB at 20% of the natural bacterial concentration. The FLB were prepared with a culture of *Brevundimonas diminuta*^1^ following Vázquez-Domínguez et al. (1999). Bottles were incubated in the tanks in the dark (covered with a black plastic bag) for 48 h at the same experimental temperature as the corresponding microcosms. To assess the bacterial and FLB abundance, samples were taken at the beginning and at the end of the grazing assay. Abundances of bacteria and FLB were assessed by epifluorescence microscopy as explained above. Natural bacteria were identified by their blue fluorescence when excited with UV radiation, while FLB were identified by their yellow–green fluorescence when excited with blue light. Control bottles showed no decrease in FLB at the end of the incubation time. Grazing rates of bacteria were obtained according to the equations of Salat and Marrasé (1994), for details see Lara et al. (2013).

To gather a large enough water volume to measure viral production and rate of lysed bacteria by viruses, we pooled together 0.5 L subsamples from each of the two triplicates for each temperature treatment submitted to the corresponding bubbling of CO_2_. We used the virus-reduction approach (Weinbauer et al., 2002; Wilhelm et al., 2002). Briefly, the 1.5-L subsamples were prefiltered through a 0.8 μm pore size cellulose filter (Whatman), and then concentrated by a spiral-wound cartridge (0.22 μm pore size, VIVAFlow 200), obtaining 50 ml of bacterial concentrate. Virus-free water was collected by filtering 0.5 L of seawater using a cartridge of 30 kDa molecular mass cutoff (VIVAFlow 200). A mixture of virus-free water (150 ml) and bacterial concentrate (50 ml) was prepared and distributed into four sterile Falcon plastic tubes. Two of these tubes were kept without any manipulations as controls, whereas in the other two, mitomycin C (1 μg mL^-1^ final concentration, Sigma) was added as the inducing agent of the lytic cycle. All Falcon tubes were incubated in the dark for 12 h inside the tanks at the same temperature as the microcosm. Samples for viral and bacterial abundances were collected at time zero and every 4 h of the incubation, fixed with glutaraldehyde (0.5% final concentration) and stored as described for viral abundance. Viruses and bacteria from the viral production experiments were counted by FACSCalibur flow cytometer (Becton and Dickinson), following Gasol and Del Giorgio (2000) and Brussaard (2004), respectively.

The number of viruses released by the bacterial cell (burst size, BS) was estimated from viral lytic production measurements, such as in Middelboe and Lyck (2002) and Wells and Deming (2006). The estimated BS ranged from 9 to 500 (168 ± 42) viruses per bacterium. These high values of BS are comparable to the ones observed by TEM in Arctic waters by Steward et al. (1996). Rate of lysed cells was determined as previously described by Weinbauer et al. (2002), and Winter et al. (2004). Briefly, an increase in viral abundance in the control tubes represents lytic viral production (LVP), and the difference between the viral increase in the mitomycin C treatments (total viral production, VP) and LVP gives the LysoVP. As part of the bacteria could be lost or increase during the bacteria concentration process, the LVP and LysoVP were multiplied by the bacterial correction factor to compare both viral production values from different incubation tubes. This factor was calculated by dividing the *in situ* bacterial concentrations by the T_0_ bacterial abundances in both viral production measurements (Winget et al., 2005) and ranged between 0.9 and 19.8. We then calculated the rate of bacteria lysed by viruses (BLV, cells mL^-1^ d^-1^) by dividing LVP by the burst size (BS), as described in Guixa-Boixereu (1997). The BLV was used to calculate bacterial losses by viruses as a percentage of the bacterial standing stock (BA). BA_BLV_ = (BLV ^∗^ 100)/BA_0_ (% d^-1^), where BA_0_ is the initial bacterial abundance *in situ*.

### Data Analysis

When necessary, data were log-transformed to meet normality and homoscedasticity assumptions of the test used. We averaged the microbial abundances from the six triplicates to describe its changes over time, since we did not find significant differences between the 2 groups of true replicates when applying a *T*-test. Then, we tested the differences of each microbial abundances (covering the whole experiment, and for each week, separately) among the three temperatures by one-way ANOVA analyses. Regression analysis was used to describe: (i) the relationships among heterotrophic microbial processes (BP, bacterial mortality due to protists and viruses, and viral lytic and lysogenic production) covering the whole range of *p*CO_2_ for each temperature individually; and (ii) the relationships among microbial parameters (processes and abundances) from the corresponding triplicates temperature treatments and CO_2_ bubbling. General linear models were used to determine the possible interactions of *p*CO_2_ and temperature on heterotrophic microbial processes. A 95% confidence intervals for the relationship between *p*CO_2_ and BP were also estimated. All analyses were performed with the JMP^TM^ and the statistical software R^©^ (Version 3.4.3, 2017).

## Results

### Dynamics of Microbial Abundances

**FIGURE 1:**
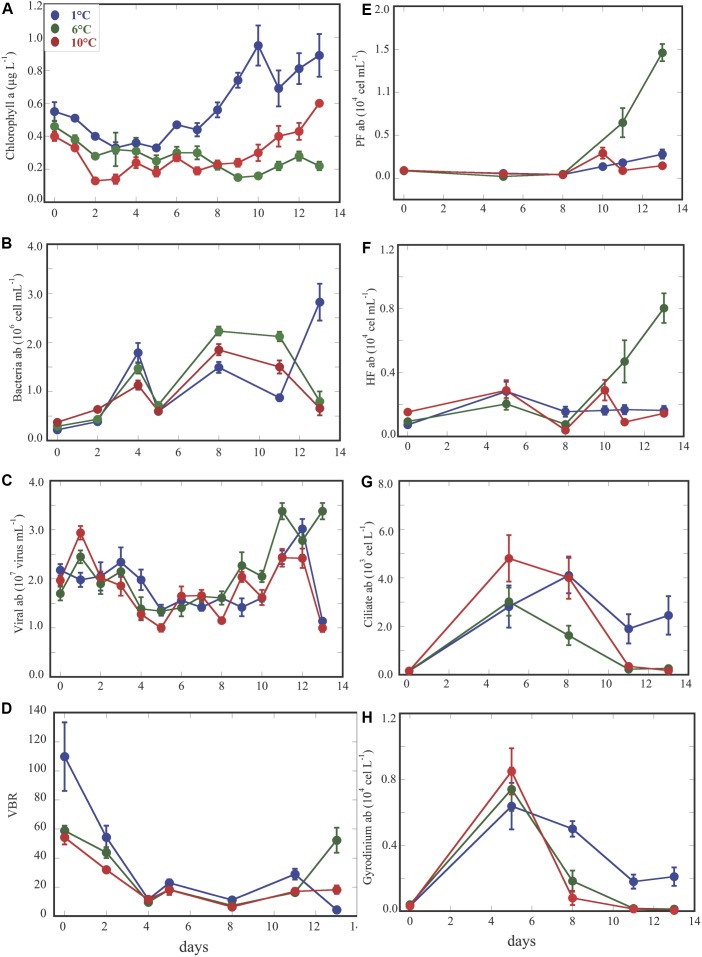
Dynamics overtime of chlorophyll *a* concentration **(A)**; bacterial abundance **(B)**; viral abundance **(C)**; VBR (viral bacteria ratio) **(D)**; phototrophic pico/nanoflagellate **(E)**; heterotrophic pico/nanoflagellate **(F)**; ciliate abundance **(G)**; *Gyrodinium* abundance **(H)**. Each point represents the averaged ±SE of six replicate values for each variable at different temperature treatments.

Abundances of microorganisms along the experiment for the three temperature treatments showed similar dynamics over the 1st week, while the main differences were detected after day 6 through the end of the experiment (Figure 1B–H and Supplementary Table S1). Chlorophyll *a* (Chla) concentration, presented significant differences across temperatures, over the first as well as in the 2nd weeks (Figure 1A and Supplementary Table S1). Bacterial abundance did not vary too much among the three temperature treatments (Figure 1B *p* > 0.05 and Supplementary Table S1), although, we detected occasionally higher values in the 6° than in the 1 and 10°C treatments after day 5 up to day 11, while opposite trends in Chl a concentration were observed in the 6 and 10°C treatments. Viral abundance displayed rather similar values across treatments for most of the duration of the experiment. Nevertheless, during the 2nd week we detected significantly higher viral abundance at 6°C (Figure 1C *p* < 0.0001 and Supplementary Table S1), whereas bacterial abundance displayed opposite high and low values at 1 and 6°C at the end of the experiment. Consequently, the increase of viruses and decrease of bacteria at 6°C, during the 2nd week, lead a VBR also significantly higher at 6°C (Figure 1D, *p* < 0.05). Phototrophic (PF) and heterotrophic (HF) pico/nanoflagellate abundances followed similar trends increasing at the end of the experiment in the 6°C treatment. Both PF and HF showed significantly higher values in the 6° than in the 10 and 1°C treatments during the 2nd week (Figure 1E,F and Supplementary Table S1). Abundances of ciliate and *Gyrodinium* sp. displayed an opposite trend than PF and HF over time. During the 2nd week, both of them showed significantly higher values at 1°C (Figure 1G, *p* < 0.005, Figure 1H, *p* < 0.001) than at the two other temperatures. Indeed, ciliates and *Gyrodinium* sp. abundances, strongly declined at 6 and 10°C at the end of the experiment, suggesting predation effects at these higher temperatures (Figure 1G,H).

In summary, our results showed that the abundances of viruses, heterotrophic and phototrophic pico/nanoflagellates increased at the 6°C treatment from the middle to the end of the experiment (Figure 1C,E,F), while Chl a was greatest at 1°C during the whole experiment (Figure 1A). And, ciliates and *Gyrodinium* sp. abundances increased rapidly at the higher temperature treatments (6 and 10°C) only to decline at the end of the experiment maintaining higher abundances only at the lowest temperature (Figure 1G,H).

### Effect of Warming and *p*CO_2_ on Heterotrophic Microbial Activities

**FIGURE 2:**
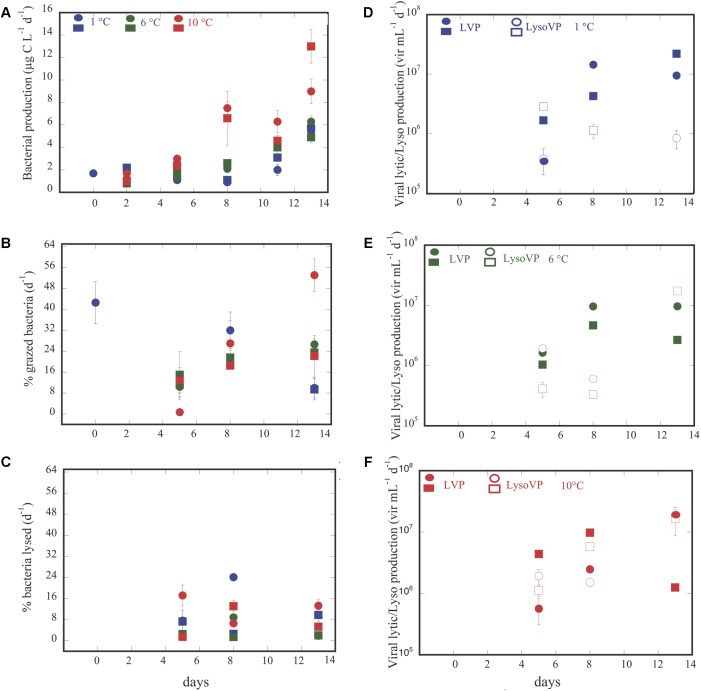
Dynamics over time of bacterial production (BP) **(A)**; bacterial grazing rates **(B)**; bacterial mortality rates due to viruses **(C)**; viral lytic and lysogenic production at 1°C **(D)**, 6°C **(E),** and 10°C **(F)**. Circles and squares, represents the averaged ± SE of each of the two groups of triplicate values for each variable submitted to different temperature treatments and exposed to the corresponding CO_2_ concentration.

**FIGURE 3:**
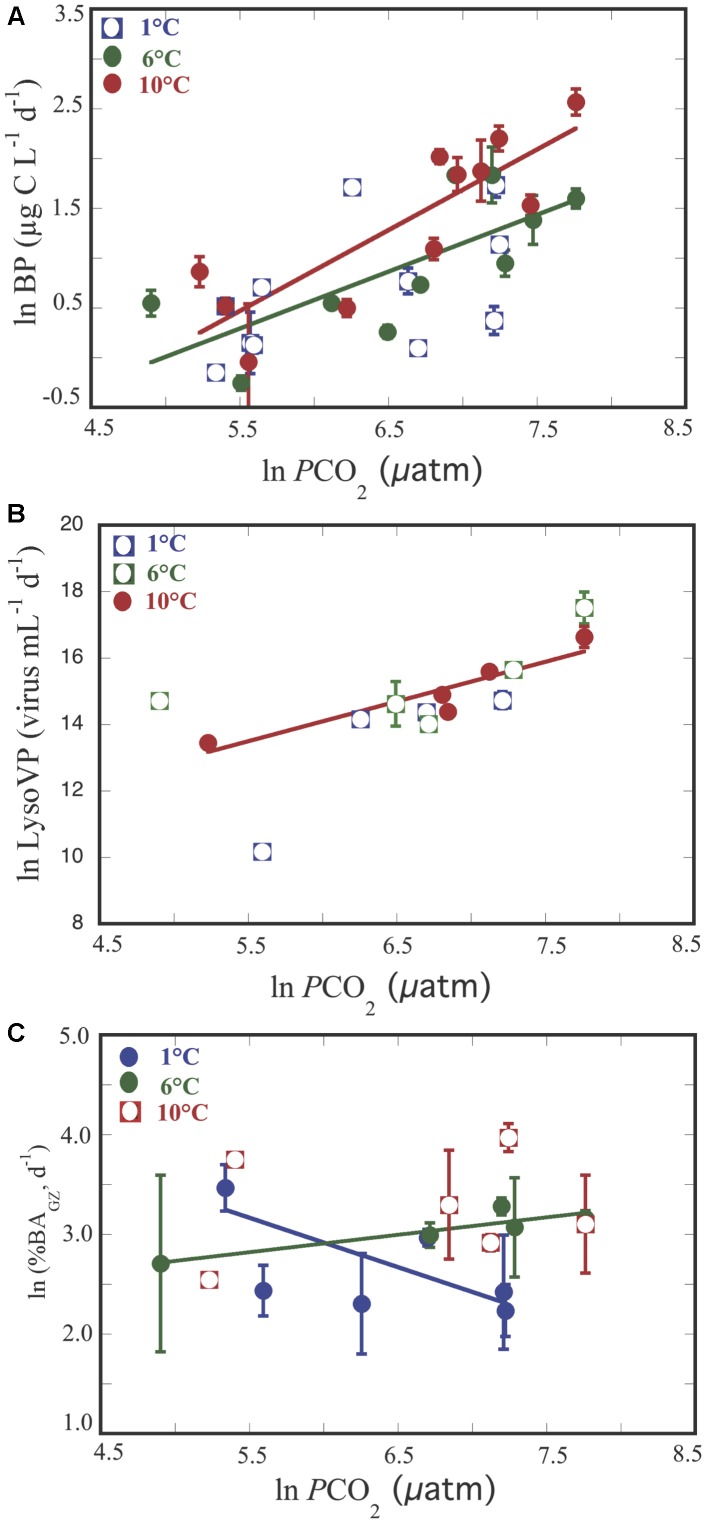
Relationship between increasing *p*CO_2_ and BP **(A)**, Lysogenic viral production (Lyso_V P_) **(B)**, percentage of bacterial losses by protists (%BA_GZ_, d^-1^) **(C)**. Each point represents the averaged ±SE of each one of the two triplicates values for each variable submitted to different temperature treatments. Empty symbols correspond to variables that did not show significant relationships, and full symbols correspond to variables that showed significant relationships for the different temperature treatments.

Bacterial production increased along the experiment for all treatments, reaching the highest values in the 10°C treatment (Figure 2A). Thus, BP varied from 0.8 μg C L^-1^ d^-1^ at temperature = 7.7°C and *p*CO_2_ = 246.6 μatm to 13.0 μg C L^-1^ d^-1^ at temperature = 10.4°C and *p*CO_2_ = 2318.3 (μatm (Supplementary Table S2), increasing with temperature (n = 32, *R*^2^ = 0.18, *p* = 0.02) and *p*CO_2_ (*n* = 33, *R*^2^ = 0.41, *p* < 0.0001). Analysis of covariance revealed that BP increased significantly with *p*CO_2_ in the 6°C, and 10°C treatments but with a steeper slope at 10°C (Figure 3A and Table 1). It was further revealed that the effect of *p*CO_2_ on BP was stronger than the effect of temperature (Table 2).

**Table 1:** Regression relationships for the natural log transformed bacterial production (BP, μg C mL^-1^ d^-1^), percentage of bacterial removed by grazing (%BA_GZ_, d^-1^), and lysogenic viral production (LysoVP, viruses mL^-1^ d^-1^) to the natural log transformed *p*CO_2_ (μatm) for each temperature treatment (1, 6, and 10°C).

	T°C	Intercept	*SE*	Slope	*SE*	*R*^2^	*p*-value
ln (BP) vs. ln (*p*CO2)	1	-2.01	1.5	0.43	0.2	0.266	0.104
	6	-2.85	1.2	0.57	0.2	0.539	**0**.**0156**^∗^
	10	-4.19	1.3	0.84	0.2	0.691	**0**.0029^**^

ln (%BA_GZ_) vs. ln (*p*CO2)	1	6.95	1.3	-0.64	0.2	0.715	**0**.**0339**^∗^
	6	1.86	0.3	0.17	0.05	0.809	**0**.**0378**^∗^
	10	0.16	6.0	0.35	0.87	0.039	0.704

ln (LysoVP) vs. ln (*p*CO2)	1	-4.29	6.8	2.74	1.0	0.773	0.121
	6	11.58	5.7	0.54	0.9	0.166	0.593
	10	6.91	1.9	1.20	0.3	0.858	**0**.**0237**^∗^


*In bold significant results. ^∗^*p* ≤ 0.05; ^∗∗^*p* ≤ 0.01.*

**Table 2:** Analysis of covariance models relating natural log transformed bacterial production (BP, μg C L^-1^ d^-1^), percentage of removed bacteria by protists (%BN_GZ_), and lysogenic viral production (LysoVP, virus mL^-1^ d^-1^) to the covariate *p*CO_2_, and temperature treatments as well as the interaction between temperature and *p*CO_2_.

Parameter estimates									

Model	Term	*n*	*R*^2^	*F*-ratio	Prob > (*F*)	Estimate	Standard error	*t*-ratio	Prob > (*t*)
ln (BP)		31	0.615	14.40	**0.0001**				
	Intercept					-3.29	0.73	-4.47	**0.0001**
	ln *p*CO_2_					0.59	0.12	5.09	**0.0001**
	Temperature					0.07	0.03	2.49	**0.0192**
	ln *p*CO_2_^∗^Temperature					0.06	0.03	1.69	0.1032

ln (%BA_GZ_)		17	0.441	3.41	**0.0499**				
	Intercept					2.78	0.74	3.78	**0.0023**
	ln *p*CO_2_					-0.03	0.12	-0.24	0.8138
	Temperature					0.05	0.03	1.64	0.1239
	ln *p*CO_2_^∗^Temperature					0.09	0.03	2.87	**0.0131**

ln (LysoVP)		13	0.756	8.26	**0.0078**				
	Intercept					1.38	2.8	0.49	0.6371
	ln *p*CO_2_					1.89	0.43	4.40	**0.0023**
	Temperature					0.07	0.09	0.87	0.4106
	ln *p*CO_2_^∗^Temperature					-0.19	0.12	-1.65	0.1382


*The parameter estimates for each model showed the intensity and the sign of pCO_2_ of these effects. Significant p-values are in bold. Only variables that presented at least one significant p-value are shown*.

Bacterial mortality rates due to protists and viruses responded opposite each other. Grazing rates (GZ) tended to increase along the experiment in the 6 and 10°C treatments (Figure 2B), while this trend was not evident for the rates of lysed bacteria (BLV, Figure 2C). Although, not statistically significant both processes showed an opposite trend (Supplementary Figure S2). Furthermore, GZ presented higher values than BLV, except for two cases at temperature = 9.9°C and at *p*CO_2_ ( = 902.3 μatm, and at temperature = 2.3°C and at *p*CO_2_ = 1382.3 μatm (Supplementary Table S2 and Supplementary Figure S2). Whereas, grazing rates and BLV were independent of *p*CO_2_ and temperature (*p* > 0.05), the percentage of bacteria removed by grazers was negatively and positively related to *p*CO_2_ at 1°C and at 6°C, respectively (Table 1 and Figure 3C), and in the covariance analyses, the interaction between *p*CO_2_ and temperature had a positive effect on the percent of bacteria grazed by protists (Table 2).

Lytic viral production (LVP) tended to increase at 1°C along the experiment (Figure 2D) and was maintained or decreased at day 13 for the other two temperatures (Figure 2E,F and Supplementary Table S2). LVP exceeded LysoVP in 12 out of 18 cases (Supplementary Table S2), from 2 up to 15 times (Figure 2D–F and Supplementary Table S2), and was independent of *p*CO_2_ and temperature (*p* > 0.05). However, in the remaining four cases LysoVP exceeded lytic viral production up to 6 times. LysoVP displayed increasing values throughout the experiment at 10°C (Figure 2F) while decreasing at 1°C (Figure 2D). In addition, LysoVP was significantly positively related to increasing *p*CO_2_ (*p*CO_2_: *n* = 13, *R*^2^ = 0.52, *p* < 0.005), but when examining the relationship between LysoVP and *p*CO_2_ at different temperatures, this was only significant for the 10°C temperature treatment (Figure 3B and Table 1). Analyses of covariance showed that LysoVP was significantly affected only by *p*CO_2_ (Table 2).

#### Relationships of the Microbial Variables

**FIGURE 4:**
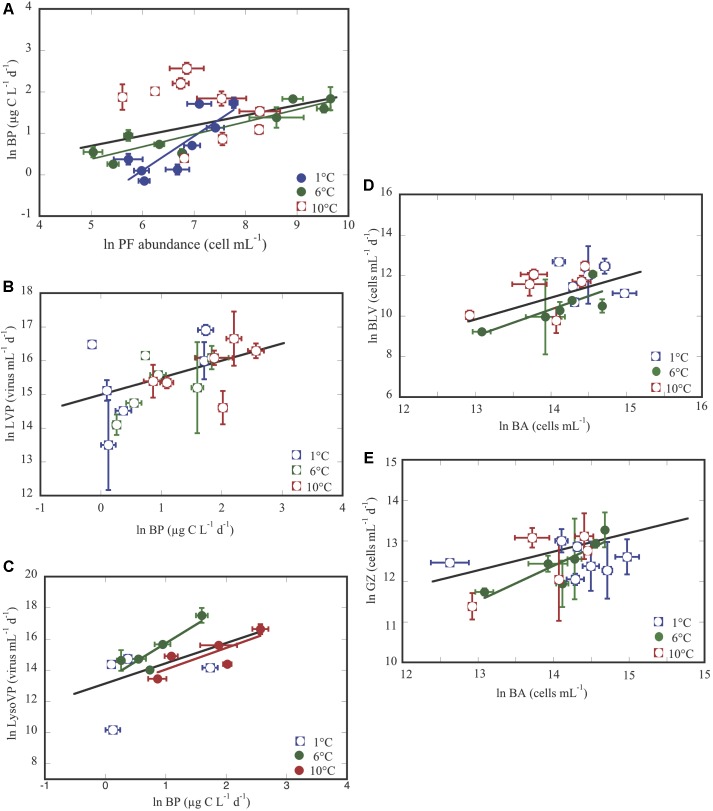
Relationship between: BP and phototrophic pico/nanoflagellates (PF) **(A)**; lytic viral production (LVP) and BP **(B)**; lysogenic viral production (LysoVP) and BP **(C)**, bacteria lysed by viruses (BLV) and bacterial abundance (BA) **(D)**; grazing rates on bacteria (GZ) and BA **(E)**. The black line corresponds to the regression line for the whole experimental data set, and color lines show the significant regressions between different variables. Each point represents the averaged ± SE of each of the two groups of triplicate values for each variable submitted to different temperature treatments and exposed to the corresponding CO_2_ concentration. Full and empty symbols represent the same as in Figure 3.

Here, we use regression analyses to describe the relationship between different microbial variables. Thus, how changes of phytoplankton affected BP, and changes of BP influence other microbial processes. So, we obtained that BP was not significantly related with Chl a concentration, that tend to decrease at the 6 and 10°C treatments with respect to the 1°C treatments, where BP increased (Figure 1B and Supplementary Table S2). Whereas, BP significantly respond to changes of abundances of phototrophic pico/nanoflagellates (PF, mainly *Pyramimonas* sp.), mainly when considering all of the temperature treatments (Figure 4A and Table 3), though significant trends were observed in the 1 and 6°C treatments, and not at all for 10°C. Notice that the 1°C treatment had the strongest response (Figure 4A and Table 3). Next, changes of viral lytic and lysogenic production were significantly related to variations of BP across temperature treatments (Figure 4B,C and Table 3). However, LysoVP was significantly related with BP in the 6°C and at 10°C treatments (Figure 4C and Table 3). Finally, bacterial mortality rates (by grazing and viral lysis) significantly responded to variations of bacterial abundance across all temperature treatments, but when considering them separately this relationship was only significant for the 6°C treatment (Figure 4D,E and Table 3).

**Table 3:** Regression analyses between ln bacterial production (BP, μg C mL^-1^ d^-1^) and ln phototrophic pico/nanoflagellate (PF, cells mL^-1^), ln lytic viral production (LVP, virus mL^-1^ d^-1^) and ln BP; ln lysogenic viral production (LysoVP, virus mL^-1^ d^-1^) and ln BP; ln grazing rates on bacteria (GZ, cells mL^-1^ d^-1^) and ln bacterial abundance (BA, cells mL^-1^); ln bacteria lysed by viruses (BLV, cells mL^-1^ d^-1^) and ln BA, covering the whole temperature (ALL) and for each temperature treatment (1, 6, and 10°C).

	T°C	Intercept	*SE*	Slope	*SE*	*n*	*R*^2^	*p*-value
ln BP vs. ln PF	ALL	-1.14	0.66	0.32	0.09	25	0.358	**0**.**0016**
	1	-4.85	1.45	0.83	0.22	9	0.678	**0**.**0064**
	6	-1.00	0.33	0.29	0.04	8	0.882	**0**.**0005**
	10	0.80	1.23	0.13	0.17	8	0.09	0.467

ln LVP vs. ln BP	ALL	14.83	0.34	0.58	0.24	18	0.264	**0**.**029**
	1	14.91	0.66	0.78	0.65	6	0.265	0.296
	6	14.53	0.58	0.80	0.51	6	0.374	0.197
	10	14.77	0.94	0.54	0.50	6	0.225	0.342

ln LysoVP vs. ln BP	ALL	13.37	0.66	1.19	0.51	14	0.310	**0**.**038**
	1	12.84	1.64	0.90	1.87	4	0.104	0.677
	6	13.36	0.70	2.37	0.75	5	0.769	<**0**.**05**
	10	12.67	1.08	1.37	0.60	5	0.636	<**0**.**05**

ln BLV vs. ln BA	ALL	-2.77	6.03	0.99	0.43	18	0.250	**0**.**035**
	1	13.27	7.79	-0.11	0.55	6	0.010	0.854
	6	-8.03	7.17	1.31	0.51	6	0.624	<**0**.**05**
	10	-4.97	8.90	1.18	0.65	6	0.454	0.142

Ln GZ vs. ln BA	ALL	6.89	2.50	0.40	0.18	18	0.240	**0**.**039**
	1	10.63	2.26	0.14	0.16	6	0.123	0.441
	6	0.21	3.62	0.87	0.26	6	0.741	**0**.**028**
	10	-20.27	27.10	2.31	1.98	6	0.254	0.308


*In bold significant results*.

## Discussion

One of the main limitations of experimental studies such as this one is to simulate projected changes that will occur over large timescales. A common concern is that short-time manipulations cannot simulate properly the effect of long-term adaptation likely occurring in natural systems and are therefore biased. However, with our experimental approach we did not pretend to mimic nature, indeed we used the experiment as a tool to help interpret how the microbial plankton could respond to environmental stressors. Furthermore, it must be noticed that, Arctic communities already experience large abrupt seasonal changes that are not experienced by many temperate oceanic communities, including rapid seasonal warming. Available sea surface temperature data from NOAA’s Climate Prediction Center^2^ corresponding to the last 2 decades for the study area (Svalbard Isfjorden) show an annual variation of 6–7°C, ranging from approximately 1–7°C, very similar to the values detected *in situ* in the North of Svalbard (-1.2–7°C) (Boras et al., 2010, ATOS-I cruise). Thus, the temperature range selected for this experiment (1–10°C) encompassed the range of temperatures experienced by the local plankton community over the year plus 2–3°C increase. Then, this increase will cover the projected range of temperatures expected in the Arctic Ocean in the coming century (ACIA, 2004).

**FIGURE 5:**
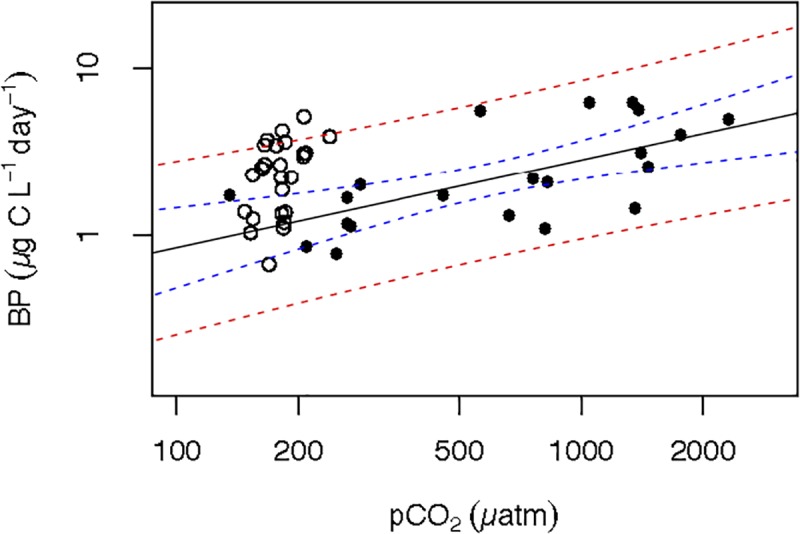
Combined BP and *p*CO_2_ data of both experiments (filled circles) and the summer ATOS-I cruise (open circles). Solid line represents the relationship of the experimental data from the 1 to 6°C temperature treatments [Log BP = –2.58 (±0.86) + 0.52 (±0.13) log *p*CO_2_; *n* = 21; *R*^2^ = 0.438; *p* < 0.003] and the dashed blue and red curves represent the 95% confidence limits for the regression equation and regression estimates, respectively.

Experiments attempting to control *p*CO_2_ in Arctic plankton communities have shown this to be an inherently difficult task (e.g., Torstensson et al., 2012; Engel et al., 2013) because the experimental subject, i.e., plankton community, contributes to variation in *p*CO_2_. Even in the field, Arctic plankton communities have been shown to drawdown *p*CO_2_ by as much as 200 ppm during the spring plankton bloom (Holding et al., 2015). Hence, active plankton growth would deplete CO_2_ faster than mass flow controllers would be able to restore target values, leading to large downward fluctuations, when high concentrations of CO_2_ were applied. Likewise, periods of high respiration, particularly under high temperature, led to elevated CO_2_ even if low concentration of CO_2_ is bubbled, along to the increase in *p*CO_2_ due – for a given mass of CO_2_ present in the water – to warming alone. This could explain the high variability in *p*CO_2_ obtained within the two groups of triplicates in each temperature treatment (Supplementary Figure S1). That, precluded to establish “low” and “high” *p*CO_2_ fixed conditions and instead to treat *p*CO_2_ as a continuous variable with a gradient-basis design following Holding et al. (2015) at concentrations ranging from 135 to 2318 μatm. Whereas, the *p*CO_2_ range observed in the Arctic Ocean across the Greenland Sea and the North of Svalbard during the ATOS-1 cruise, was between 134 and 260 μatm (Alvarez, personal communication). Nevertheless, when examining the relationship between BP and *p*CO_2_ from data derived experimentally and *in situ* surface *p*CO_2_ and BP (Alvarez, unpublished results, Boras et al., 2010, respectively) within similar temperature ranges (≤7.0°C), both *in situ* and experimental data fall within the confidence limits of the experimentally derived relationship between BP and *p*CO_2_ (Figure 5). The consistency of this relationship considering both scenarios, revealed that CO_2_, directly or indirectly through phytoplankton, could have a strong influence on BP.

Our experimental data show that warming (6°C and 10°C) at increasing *p*CO_2_ enhances BP in these Arctic waters. Indeed, we think that variations of primary producers subjected to increasing *p*CO_2_ and temperature probably contributed to changes in BP. As in the same experimental set up Holding et al. (2015) found that the effect of fertilization of CO_2_ did not increase further, beyond 6°C, with a steeper slope at 1°C (almost twice) than at 6°C. This is, that the higher temperatures of 10°C promoted a decrease of net primary production and increased respiration (Holding et al., 2013), due to phytoplankton taxa shifting from larger to smaller cell size cells (Coello-Camba et al., 2014) and the associated release of dissolved organic matter, favoring BP, as is shown in Morán et al. (2006). This fact, is also reflected by a lack of relationship at 10°C between BP and abundance of phototrophic pico/nanoflagellates (Figure 4A, i.e., *Pyramimonas* sp., Prasinophytes class; Coello-Camba et al., 2014). The PF are the main representative primary producers at the time of the experiment and significantly related with BP at 1 and 6°C (Figure 4A). The small Prasinophytes achieved high abundances at 6°C, in detriment of other taxa (Coello-Camba et al., 2014), only to decrease again at 10°C (Figure 1E), as was also described in a previous experiment by Lara et al. (2013) in Svalbard, where the most abundant genus was *Micromonas* sp. This fact agrees with the finding of Lovejoy et al. (2007), and Vaqué et al. (2008) when in the Arctic Ocean, blooms of picoprasinophytes and/or *Micromonas* were observed during spring and summer. With respect to the increase of BP, Davidson et al. (2016) showed that the Antarctic heterotrophic bacterial communities were more active and diverse at higher CO_2_ concentrations, which was attributed to the increase in compounds from phytoplankton for bacterial to growth (Piontek et al., 2013). Also, Lindh et al. (2013) in mesocosms experiment done in the Baltic Sea, and Keys et al. (2018) in the English-channel found that the increase of BP derived from changes of bacterial communities, favoring different phylogenetic types when subjected to warming and acidification.

Next, changes in BP production in these Arctic waters, were expected to be propagated to other microbial processes, such as viral lytic production and lysogeny, and bacterial mortality rates. Although, we are not aware of any other experimental study combining the effect of increasing *p*CO_2_ together with warming on bacterial losses by protists or viruses to compare with, there are several studies describing these effects of warming and/or *p*CO_2_ separately on these bacterial mortality. During our experiments grazing rates at different temperature treatments slightly increased after the 5th day at 6°C (Figure 2B). This effect of warming on bacterial mortality was also observed as in experimental as well as *in situ* samples of Antarctic marine communities (Vaqué et al., 2009; Sarmento et al., 2011) and Arctic waters (Lara et al., 2013). On the other hand, it is also described that low *in situ* temperature in high latitudes constrained the growth of heterotrophic protists on bacteria and phytoplankton (Rose and Caron, 2007). Furthermore, we also observed that bacterial mortality due to grazing was almost always higher than that caused by viral lysis, mainly at 6°C (Figure 2B,C). These results agree with other studies both, *in situ* where Maranger et al. (2015) reported a stronger temperature-control on grazing rates compared to bacterial lysis rates in a pan-arctic study, as well as in warming controlled experiments (Lara et al., 2013). In contrast, Vaqué et al. (2017) showed that BP and mortality rates due to viruses were more sensitive to temperature than grazing activity in Antarctic regions.

Regarding the effect of *p*CO_2_ on viral lysis rates, Larsen et al. (2008) and Brussaard et al. (2013) reported that the activity of virioplankton on phytoplankton did not show a clear response to increasing *p*CO_2_ in an Arctic mesocosm experiment. Danovaro et al. (2011) suggested that viral abundance responses maybe mediated by the effects of pH and consequently to *p*CO_2_ on the host organisms: bacteria, archaea, protists and metazoan. Indeed, the rate of lysed bacteria in our experiment seems to be mainly dependent on the host communities (Figure 2D) rather than directly on *p*CO_2_ and/or temperature. While percentage of removed bacteria by protists seems to be affected by both temperature and *p*CO_2_ (Table 2).

It is described that lysogeny should be the dominant viral life strategy in oligotrophic systems (Jiang and Paul, 1994; Danovaro et al., 2011, and references therein) as is the case in Isfjorden at the sampling time, which is characterized by low temperature (1.4°C), low phytoplankton Chla (0.6 ± 0.06 μg L^-1^), low bacterial abundance (2.2 ± 0.4^∗^10^5^ cell mL^-1^) and low BP (1.7 μg C L^-1^ d^-1^) (Figure 1A,B and Supplementary Table S2). However, our results showed more lysis at low than at high temperature treatments. Indeed, in our experiment lysogeny significantly increased as *p*CO_2_ increased at 10°C in parallel with BP (Table 1 and Figure 2A,F). In addition, lysogeny was significantly related with BP at 6 and at 10°C temperature treatments, and not at all at 1°C (Figure 4C and Table 3). These results agree with the proposed Piggyback-the–Winner model by Knowles et al. (2016) where lysogeny should be even more successful than the lytic cycle when bacterial hosts are growing well. Here, we suspected, as is shown in Lindh et al. (2013) that warming and increased CO_2_ will promote the selection of specific bacterial communities, responsible for high BP. And, Knowles et al. (2016) assume that viruses “exploit” their hosts (that are presumably highly active bacteria) through lysogeny instead of killing them, making it advantageous for both of them; the prophage is propagated to the new bacteria generation, protects the host for a new viral infection (Levin and Lenski, 1983) and predation by grazers (Brüssow, 2007). Furthermore, in the 1°C treatment, where we detected lower BP than at higher temperatures, the lytic cycle was more important than lysogeny (Figure 2A,D–F). The observations of more cases of lysogeny at warmer than at the lower temperature treatments, and its increase at increasing BP suggests a decreasing flow of bacterial carbon entering in a dissolved phase via the viral shunt (lysed bacteria), thereby favoring the microbial loop (grazing on bacteria). This indicates an enhancement of heterotrophic biomass and processes relative to phototrophic processes with warming, as previously reported, based on temperature manipulations described by Holding et al. (2013) and Lara et al. (2013).

## Conclusion

Our experimental results provide evidence that possible future changes in *p*CO_2_ and temperature in the Arctic Ocean may lead to increased bacteria production, probably triggered by changes in primary producers that will be propagated to other microbial activities. The increasing *p*CO_2_ and temperature is additive in the case of BP, and their effect on the relative flow of bacterial carbon through grazers (protists) depends on their interaction. Next, the increase of BP enhanced lysogeny more than viral lysis, which will create changes in the carbon fluxes within the microbial food web, evidenced by the higher bacteria mortality rates due to protists than to viruses. While the current debate on the responses of the Arctic to the expected changes of temperature and *p*CO_2_ are focused only on primary production, our experiments suggest that increased heterotrophic BP, and decreased phytoplankton biomass, in a warmer more acidic Arctic, may lead to a reduced net community production, which will be translated in a weakening CO_2_ sink capacity of the Arctic plankton community. However, there is a need of more experimental and field studies that will encompasses different seasonal situations and long time period to corroborate these poor expectations.

## Author Contributions

DV and EL designed and coordinated this study, collected samples for viruses, bacteria and protists, performed mortality experiments, analyzed the results, and wrote the manuscript. JA designed and implemented the whole experimental set up, responsible for bacterial abundance and production measurement. JH helped in the data analyses and in the elaboration of the manuscript. ES collaborated in the setup of the experiments, in the daily sampling, and in the lab. IH helped in the implementation of the CO_2_ bubbling system. AC-C collaborated in sampling, in the chlorophyll *a* determination and phytoplankton cells count. MA contributed with CO_2_ data from ATOS-I cruise. SA coordinated the phytoplankton study and sampling, its analysis and identification. PW was the PI of the ATP project, making possible the use of facilities in UNIS (Sbalvard) to carry out the experiments, and added valuable advice to the study. CD coordinator of the ATP project (CSIC), provided a creative environment and added constructive criticisms throughout the study revising and editing the manuscript. All authors commented and discussed the obtained results, and suggested improvements on the manuscript.

## Conflict of Interest Statement

The authors declare that the research was conducted in the absence of any commercial or financial relationships that could be construed as a potential conflict of interest.

## Footnotes

1 http://www.uv.es/cect

2 https://www.cpc.ncep.noaa.gov/products/CFSv2/CFSv2seasonal.shtml

## References

[B1] ACIA. (2004). *Impacts of a Warming Arctic: Arctic Climate Impact Assessment.* Cambridge: Cambridge University Press.

[B2] Bates N. R. Moran S. B. Hansell D. A. Mathis J. T. (2006). An increasing CO_2_ sink in the arctic ocean due to sea-ice loss. *Geoph. Res. Lett.* 33:L23609.

[B3] Beardall J. Stojkovic S. Larsen S. (2009). Living in a high CO_2_ world: impacts of global climate change on marine phytoplankton. *Plant. Ecol. Div.* 2 191–205. 10.1080/17550870903271363

[B4] Boras J. A. Sala M. M. Arrieta J. M. Sà E. L. Felipe J. Agustí S. (2010). Effect of ice melting on bacterial carbon fluxes channelled by viruses and protists in the Arctic Ocean. *Pol. Biol.* 33 1695–1707. 10.1007/s00300-010-0798-8

[B5] Brussaard C. P. D. (2004). Optimization of procedures for counting viruses by flow cytometry. *App. Environ. Microbiol.* 70 1506–1513. 10.1128/AEM.70.3.1506-1513.2004 PMC368280 15006772

[B6] Brussaard C. P. D. Noordeloos A. A. M. Witte H. Collenteur M. C. J. Schulz K. Ludwig A. (2013). Arctic microbial community dynamics influenced by elevated CO_2_ levels. *Biogeoscience* 10 719–731. 10.5194/bg-10-719-2013

[B7] Brüssow H. (2007). Bacteria between protists and phages: from antipredation strategies to the evolution of pathogenicity. *Mol. Microbiol.* 65 583–589. 10.1111/j.1365-2958.2007.05826.x 17608793

[B8] Coello-Camba A. Agustí S. Holding J. Arrieta J. M. Duarte C. M. (2014). Interactive effect of temperature and CO_2_ increase in arctic phytoplankton. *Front. Mar. Sci.* 1:49.

[B9] Coello-Camba A. Agustí S. Vaqué D. Holding J. Arrieta J. Wassmann P. (2015). Experimental assessment of temperature thresholds for arctic phytoplankton communities. *Est. Coasts.* 38 873–885. 10.1007/s12237-014-9849-7

[B10] Danovaro R. Corinaldesi C. Dell’Anno A. Fuhrman J. A. Middelburg J. J. Noble R. T. (2011). Marine viruses and global climate change. *FEMS Microbiol. Rev.* 35 993–1034. 10.1111/j.1574-6976.2010.00258.x 21204862

[B11] Davidson A. T. McKinlay J. Westwood K. Thomson P. G. van den Enden R. de Salas M. (2016). Enhanced CO_2_ concentrations change the structure of Antarctic marine microbial communities. *Mar. Prog. Oceanog.* 552 93–113. 10.3354/meps11742

[B12] Dickson A. G. Sabine C. L. Christian J. R. (eds) (2007). *Guide to Best Practices for Ocean CO_2_ Measurements*, Vol. 3 Sidney, BC: PICES Special Publication

[B13] Engel A. Borchard C. Piontek J. Schulz K. G. Riebesell U. Bellerby R. (2013). CO_2_ increases 14C primary production in an arctic plankton community. *Biogeoscience* 10 1291–1308. 10.5194/bg-10-1291-2013

[B14] Gasol J. M. Del Giorgio P. A. (2000). Using flow cytometry for counting natural planktonic bacteria and understanding the structure of planktonic bacterial communities. *Sci. Mar.* 64 197–224. 10.3989/scimar.2000.64n2197

[B15] Guixa-Boixereu N. (1997). *Abundància I Dinàmica Dels Virus En Ecosistemes Planctònics.* Doctoral Thesis, Universitat de Barcelona, Barcelona.

[B16] Holding J. M. Duarte C. M. Arrieta J. M. Vaquer-Sunyer R. Coello-Camba A. Wassmann P. F. (2013). Experimentally determined temperature thresholds for arctic plankton community metabolism. *Biogeoscience* 10 357–370. 10.5194/bg-10-357-2013

[B17] Holding J. M. Duarte C. M. Sanz-Martin M. Mesa E. Arrieta J. M. Chierici M. (2015). Temperature dependence of CO_2_-enhanced primary production in the european arctic ocean. *Nat. Commun.* 5 1079–1082. 10.1038/nclimate2768

[B18] Intergovernmental Panel on Climate Change [IPCC]. (2014). *Contribution of Working Group III. Chapter V. Drivers Trends and Mitigation.* Available at https://www.ipcc.ch/site/assets/uploads/2018/02/ipcc_wg3-ar5_chapter5.pdf

[B19] Jiang S. C. Paul J. H. (1994). Seasonal and diel abundance of viruses and occurrence of lysogeny/bacteriocinogeny in the marine environment. *Mar. Ecol. Prog. Ser.* 104 163–172. 10.3354/meps104163

[B20] Keys M. Tilstone G. Findlay H. S. Widdicombe C. E. Lawson T. (2018). Effects of elevated CO_2_ and temperature on phytoplankton community biomass, species composition and photosynthesis during an experimentally induced autumn bloom in the western english channel. *Biogeoscience* 15 3203–3222. 10.5194/bg-15-3203-2018

[B21] Kirchman D. Knees E. Hodson R. (1985). Leucine incorporation and its potential as a measure of protein-synthesis by bacteria in natural aquatic systems. *Appl. Environ. Microbiol.* 49 599–607. 3994368 10.1128/aem.49.3.599-607.1985 PMC373556

[B22] Kirchman D. L. (1993). “Leucine incorporation as a measure of biomass production by 96 heterotrophic bacteria,” in *Handbook of Methods of Aquatic Microbial Ecology*, eds Kemp P. F. Sherr B. F. Sherr E. B. Cole J. J. (Boca Raton: Lewis Publishers), 509–512.

[B23] Knowles B. Silveira C. B. Bailey A. B. Barott K. Cantu V. A. Cobián-Güemes A. G. (2016). Lytic to temperate switching of viral communities. *Nature* 531 4666–4670. 10.1038/nature17193 26982729

[B24] Lara E. Arrieta J. M. Garcia-Zarandona I. Boras J. A. Duarte C. M. Agustí S. (2013). Experimental evaluation of the warming effect on viral, bacterial and protistan communities in two contrasting arctic systems. *Aquat. Microb. Ecol.* 70 17–32. 10.3354/ame01636

[B25] Larsen J. B. Larsen A. Thyrhaug R. Bratbak G. Sandaa R. A. (2008). Response of marine viral populations to a nutrient induced phytoplankton bloom at different pCO_2_ levels. *Biogeoscience* 5 523–533. 10.5194/bg-5-523-2008

[B26] Levin B. R. Lenski R. E. (1983). “Coevolution in bacteria and their viruses and plasmids,” in *Coevolution*, eds Futuyma D. J. Slatkin M. (Sunderland: Sinauer).

[B27] Lindh M. V. Riemann L. Baltar F. Romero-Oliva C. Salomon P. S. Granéli E. (2013). Consequences of increased temperature and acidification on bacterioplankton community composition during a mesocosm spring bloom in the Baltic Sea. *Env. Microbiol. Rep.* 5 252–262. 10.1111/1758-2229.12009 23584969

[B28] Lovejoy C. Vincent W. Bonilla S. Roy S. Martineau M.-J. Terrado M. (2007). Distribution, phylogeny, and growth of cold adapted picoprasinophytes in arctic sea. *J. Phycol.* 43 78–89. 10.1111/j.1529-8817.2006.00310.x

[B29] Maranger R. Vaqué D. Nguyen D. Hébert M. P. Lara E. (2015). Pan-Arctic patterns of planktonic heterotrophic microbial abundance and processes: controlling factors and potential impacts of warming. *Progr. Oceanogr.* 139 221–232. 10.1016/j.pocean.2015.07.006

[B30] Marie D. Brussaard C. P. D. Thyrhaug R. Bratbak G. Vaulot D. (1999). Enumeration of marine viruses in culture and natural samples by flow cytometry. *Appl. Environ. Microbiol.* 65 45–52. 9872758 10.1128/aem.65.1.45-52.1999 PMC90981

[B31] Meehl G. A. Stocker T. F. Collins W. D. Friedlingstein P. Gaye A. T. Gregory J. M. (2007). “Global climate projections,” in *Climate Change 2007: The Physical Science Basis. Contribution of Working Group I to the Fourth Assessment Report of the Intergovernmental Panel on Climate Change*, eds Solomon S. Qin D. Manning M. Chen Z. Marquis M. Averyt K. B. (Cambridge: Cambridge University Press), 749–845.

[B32] Middelboe M. Lyck P. G. (2002). Regeneration of dissolved organic matter by viral lysis in marine microbial communities. *Aquat. Microb. Ecol.* 27 187–194. 10.3354/ame027187

[B33] Morán X. A. G. Sebastián M. Pedrós-Alió C. Estrada M. (2006). Response of southern ocean phytoplankton and bacterioplankton production to short-term experimental warming. *Limnol. Oceanogr.* 51 1791–1800. 10.4319/lo.2006.51.4.1791

[B34] Parkinson C. L. Comiso J. C. (2013). On the 2012 record low arctic sea ice cover: Combined impact of preconditioning and an august storm. *Geoph. Res. Lett.* 40 1356–1361. 10.1002/grl.50349

[B35] Parsons T. R. Maita Y. Lalli C. M. (1984). *A Manual of Chemical and Biological Methods for Seawater Analysis.* Oxford: Pergamon Press.

[B36] Pierrot D. Lewis E. Wallace D. W. R. (2006). *MS Excel Program Developed for CO_2_ System Calculations. ORNL/CDIAC-105a.* Oak Ridge: Carbon Dioxide Information Analysis Center.

[B37] Piontek J. Borchard C. Sperling M. Schulz K. G. Riebesell U. Engel A. (2013). Response of bacterioplankton activity in an Arctic fjord system to elevated pCO_2_: results from a mesocosm perturbation study. *Biogeoscience* 10 297–314. 10.5194/bg-10-297-2013

[B38] Popova E. E. Yool A. Aksenov Y. Coward A. C. Anderson T. R. (2014). Regional variability of acidification in the Arctic: a sea of contrasts. *Biogeoscience* 11 293–308. 10.5194/bg-11-293-2014

[B39] Porter K. G. Feig Y. S. (1980). The use of DAPI for identifying and counting aquatic microflora. *Limnol. Oceanogr.* 25 943–948. 10.4319/lo.1980.25.5.0943

[B40] Rose J. M. Caron D. A. (2007). Does low temperature constrain the growth rates of heterotrophic protists? Evidence and implications for algal blooms in cold waters. *Limol. Oceanog.* 52 886–895. 10.4319/lo.2007.52.2.0886

[B41] Salat J. Marrasé C. (1994). Exponential and linear estimations of grazing on bacteria: effects of changes in the proportion of marked cells. *Mar. Ecol. Prog. Ser.* 104 205–209. 10.3354/meps104205

[B42] Sanz-Martin M. Chierici M. Mesa E. Carrillo-de-Albornoz P. Delgado-Huertas A. Agusti S. (2018). Episodic arctic CO_2_ limitation in the west svalbard shelf. *Front. Mar. Sci.* 5:221 10.3389/fmars.2018.00221

[B43] Sarmento H. Montoya J. M. Vázquez-Domínguez E. Vaque D. Gasol J. M. (2011). Warming effects on marine microbial food web processes: how far can we go when it comes to predictions? *Phil. Trans. R. Soc. B Biolog. Sci.* 365 2137–2149. 10.1098/rstb.2010.0045 20513721 PMC2880134

[B44] Shadwick E. H. Trull T. W. Thomas H. Gibson J. A. E. (2013). Vulnerability of polar oceans to anthropogenic acidification: comparison of arctic and antarctic seasonal cycles. *Sci. Rep.* 3:2339. 10.1038/srep02339 23903871 PMC3730166

[B45] Sherr B. F. Sherr E. B. Fallon R. D. (1987). Use of monodispersed, fluorescently labeled bacteria to estimate in situ protozoan bacterivory. *App. Environ. Microbiol.* 53 958–965. 16347355 10.1128/aem.53.5.958-965.1987 PMC203794

[B46] Sieracki M. E. Johnson P. W. Sieburth J. M. (1985). Detection, enumeration, and sizing of planktonic bacteria by image-analyzed epifluorescence microscopy. *App. Environ. Microbiol.* 49 799–810. 2408564 10.1128/aem.49.4.799-810.1985 PMC238449

[B47] Smith D. C. Azam F. (1992). A simple, economical method for measuring bacterial protein synthesis rates in seawater using 3H-leucine. *Mar. Microb. Food Webs* 6 107–114.

[B48] Steward G. F. Smith D. C. Azam F. (1996). Abundance and production of bacteria and viruses in the bering and chukchi seas. *Mar. Ecol. Prog. Ser.* 131 287–300. 10.3354/meps131287

[B49] Takahashi T. Sutherland S. C. Sweeney C. Poisson A. Metzl N. Tilbrook B. (2002). Global sea–air CO_2_ flux based on climatological surface ocean pCO_2_, and seasonal biological and temperature effects. *Deep Sea Res. Part II Top. Stud. Oceanogr.* 49 1601–1622. 10.1016/S0967-0645(02)00003-6

[B50] Torstensson A. Chierici M. Wulff A. (2012). The influence of increased temperature and carbon dioxide levels on the benthic/sea ice diatom navicula directa. *Polar Biol.* 35 205–214. 10.1007/s00300-011-1056-4

[B51] United States Environmental Protection Agency [USEPA] (2013). *Future of Climate Change.* Available at https://19january2017snapshot.epa.gov/climate-change-science/future-climate-change_.html.

[B52] Vaqué D. Boras J. Torrent-Llagostera F. Agustí S. Arrieta J. M. Lara E. (2017). Viruses and protists induced-mortality of prokaryotes around the antarctic peninsula during the austral summer. *Front. Microbiol.* 8:241. 10.3389/fmicb.2017.00241 28303119 PMC5332362

[B53] Vaqué D. Guadayol O. Peters F. Felipe J. Angel-Ripoll L. Terrado R. (2008). Seasonal changes in planktonic bacterivory rates under the ice-covered coastal arctic ocean. *Limnol. Oceanog.* 53 2427–2438. 10.4319/lo.2008.53.6.2427

[B54] Vaqué D. Guadayol O. Peters F. Felipe J. Malits A. Pedrós-Alió C. (2009). Differential response of grazing and bacterial heterotrophic production to experimental warming in antarctic waters. *Aquat. Microb. Ecol.* 54 101–112. 10.3354/ame01259

[B55] Vaquer-Sunyer R. Duarte C. M. Holding J. Regaudie-de-Gioux A. García-Corral L. S. Reigstad M. (2013). Seasonal patterns in Arctic planktonic metabolism (fram strait – svalbard region). *Biogeoscience* 10 1451–1469. 10.5194/bg-10-1451-2013

[B56] Vázquez-Domínguez E. Peters F. Gasol J. M. Vaqué D. (1999). Measuring the grazing losses of picoplankton: methodological improvements in the use of fluorescently labeled tracers combined with flow cytometry. *Aquat. Microb. Ecol.* 20 119–128. 10.3354/ame020119

[B57] Weinbauer M. G. Winter C. Hofle M. G. (2002). Reconsidering transmission electron microscopy based estimates of viral infection of bacterio-plankt,n using conversion factors derived from natural communities. *Aquat. Microb. Ecol.* 27 103–110. 10.3354/ame027103

[B58] Wells L. E. Deming J. W. (2006). Significance of bacterivory and viral lysis in bottom waters of Franklin Bay, Canadian Arctic, during winter. *Aquat. Microb. Ecol.* 43 209–221. 10.3354/ame043209

[B59] Wilhelm S. W. Brigden S. M. Suttle C. A. (2002). A dilution technique for the direct measurement of viral production: a comparison in stratified and tidally mixed coastal waters. *Microb. Ecol.* 43 168–173. 10.1007/s00248-001-1021-9 11984638

[B60] Winget D. M. Williamson K. E. Helton R. R. Wommack K. E. (2005). Tangential flow diafiltration: an improved technique for estimation of virioplankton production. *Aquat. Microb. Ecol.* 41 221–232. 10.3354/ame041221

[B61] Winter C. Herndl G. J. Weinbauer M. G. (2004). Diel cycles in viral infection of bacterioplankton in the north sea. *Aquat. Microb. Ecol.* 35 207–216. 10.3354/ame035207

[B62] Wohlers J. Engel A. Zölnner E. Breithaupt P. Jürgens K. Hoppe H.-G. (2009). Changes in biogenic carbon flow in response to sea surface warming. *Proc. Natl. Acad. Sci. U.S.A.* 106 7067–7072. 10.1073/pnas.0812743106 19359482 PMC2678444

