# Supplementary material for: Warming and CO2 Enhance Arctic Heterotrophic Microbial Activity
Source: Front Microbiol. 2019 Mar 20;10:494. doi: 10.3389/fmicb.2019.00494 (PMC6436474; doi:10.3389/fmicb.2019.00494)
Supplement: Supplementary file 1 [file Table_1.DOCX]

**Table 1SM.** Results of the ANOVA test for abundances of microorganisms along the first and the second week of the experiment, among different temperature treatments. When the ANOVA test was significant, differences between each two temperatures were assessed with Post hocTukey test. Significant values are indicated with p<0.05. ns: no significant, n = number of data, F: Fisher value, p: level of significance. Chl a – chlorophyll a concentration, BA- bacterial abundance, VA- viral abundance, VBR- viral bacteria ratio, PF-phototrophic pico/nanoflagellate abundance, HF– heterotrophic pico/nanoflagellate abundance, Cil – Ciliate abundance, Gyr – *Gyrodinium* sp. abundance.

First week (0-7 days) Second week (8-13 days)

| **VAR/temp** | **n** | **F** | ***p*** |  | **VAR/temp** | **n** | **F** | ***p*** |
| --- | --- | --- | --- | --- | --- | --- | --- | --- |
|  |  |  |  |  |  |  |  |  |
| Chl a ALL | 144 | 31.39 | <0.0001 |  | Chl a ALL | 107 | 72.09 | <0.0001 |
| 1 – 6 °C |  |  | 0.0002 |  | 1 – 6 °C |  |  | <0.0001 |
| 1 – 10° C |  |  | <0.0001 |  | 1 – 10° C |  |  | <0.0001 |
| 6 – 10° C |  |  | 0.0008 |  | 6 – 10° C |  |  | 0.0009 |
|  |  |  |  |  |  |  |  |  |
| BA ALL | 65 | 0.28 | ns |  | BA ALL | 53 | 1.08 | ns |
|  |  |  |  |  |  |  |  |  |
| VA ALL | 144 | 0.44 | ns |  | VA ALL | 108 | 12.93 | <0.0001 |
| 1 – 6 °C |  |  | ns |  | 1 – 6 °C |  |  | 0.0002 |
| 1 – 10° C |  |  | ns |  | 1 – 10° C |  |  | ns |
| 6 – 10° C |  |  | ns |  | 6 – 10° C |  |  | <0.0001 |
|  |  |  |  |  |  |  |  |  |
| VBR ALL | 65 | 1.44 | ns |  | VBR ALL | 53 | 3.37 | 0.0424 |
| 1 – 6 °C |  |  | ns |  | 1 – 6 °C |  |  | ns |
| 1 – 10° C |  |  | ns |  | 1 – 10° C |  |  | ns |
| 6 – 10° C |  |  | ns |  | 6 – 10° C |  |  | <0.05 |
|  |  |  |  |  |  |  |  |  |
| PF ALL | 36 | 1.46 | ns |  | PF ALL | 66 | 9.73 | 0.0002 |
| 1 – 6 °C |  |  | ns |  | 1 – 6 °C |  |  | 0.0001 |
| 1 – 10° C |  |  | ns |  | 1 – 10° C |  |  | ns |
| 6 – 10° C |  |  | ns |  | 6 – 10° C |  |  | 0.0095 |
|  |  |  |  |  |  |  |  |  |
| HF ALL | 36 | 1.06 | ns |  | HF ALL | 66 | 13.02 | <0.0001 |
| 1 – 6 °C |  |  | ns |  | 1 – 6 °C |  |  | 0.0001 |
| 1 – 10° C |  |  | ns |  | 1 – 10° C |  |  | ns |
| 6 – 10° C |  |  | ns |  | 6 – 10° C |  |  | <0.0001 |
|  |  |  |  |  |  |  |  |  |
| Ciliate ALL | 35 | 0.79 | ns |  | Ciliate ALL | 46 | 6.56 | 0.003 |
| 1 – 6 °C |  |  | ns |  | 1 – 6 °C |  |  | 0.0025 |
| 1 – 10° C |  |  | ns |  | 1 – 10° C |  |  | ns |
| 6 – 10° C |  |  | ns |  | 6 – 10° C |  |  | ns |
|  |  |  |  |  |  |  |  |  |
| Gyr ALL | 31 | 0.36 | ns |  | Gyr ALL | 43 | 13.36 | <0.0001 |
| 1 – 6 °C |  |  | ns |  | 1 – 6 °C |  |  | 0.0003 |
| 1 – 10° C |  |  | ns |  | 1 – 10° C |  |  | 0.0003 |
| 6 – 10° C |  |  | ns |  | 6 – 10° C |  |  | ns |

**Table 2SM**. Results of the two groups of triplicated microcosms experiment organized by day, temperature treatment (Temp ± SE, °C), partial pressure of CO_2_ (*p*CO_2_, µatm), bacterial production (BP ± SE, µg C L^-1^ d^-1^), grazing rates on bacteria (GZ ± SE, 10^5^ cells mL^-1^ d^-1^), percentage of removed bacteria by protists (%BA_GZ_ ± SE), rate of bacteria lysed by viruses (BLV ± SE, 10^5^ cells mL^-1^ d^-1^), percentage of removed bacteria by viruses (%BA_BLV_ ± SE), percentage of lytic bacterial production (%LVP ± SE), and percentage of lysogenic viral production (% LysoVP ± SE) measured overtime.

| **Days** | **Temp** | ***p*CO_2_** | **BP** | **GZ** | **%BA_GZ_** | **BLV** | **%BA_BLV_** | **%LVP** | **%LysoVP** |
| --- | --- | --- | --- | --- | --- | --- | --- | --- | --- |
|  |  |  |  |  |  |  |  |  |  |
| 0 | 1.41 | 222.4 | 1.7 ±0.1 | 2.6 ± 0.1 | 42.6 ± 0.8 | - | - | - | - |
|  |  |  |  |  |  |  |  |  |  |
| 2 | 1.94±0.0 | 262.7 | 1.2 ± 0.4 | - | - | - | - | - | - |
| 3 | 1.72±0.2 | 207.9 | - | - | - | - | - | - | - |
| 4 | 1.95±0.1 | 286.2 | - | - | - | - | - | - | - |
| 5 | 1.64±0.0 | 268.9 | 1.1 ± 0.2 | 1.7 ± 0.4 | 11.5 ± 3.2 | 0.9 ± 0.0 | 7.6 ± 0.0 | 50.0 ± 35.4 | 50.0 ± 35.4 |
| 6 | 1.61±0.1 | 523.4 | - | - | - | - | - | - | - |
| 7 | 1.51±0.1 | 561.0 | - | - | - | - | - | - | - |
| 8 | 1.23±0.1 | 208.2 | 0.9 ± 0.1 | 4.4 ± 1.3 | 32.0 ± 7.0 | 3.2 ± 0.3 | 24.1 ± 0.2 | 100.0 | 0.0 |
| 9 | 1.63±0.3 | 180.2 | - | - | - | - | - | - | - |
| 10 | 1.85±0.2 | 398.8 | - | - | - | - | - | - | - |
| 11 | 1.74±0.3 | 283.7 | 2.0 ± 0.5 | - | - | - | - | - | - |
| 12 | 2.50±0.0 | 553.8 | - | - | - | - | - | - | - |
| 13 | 2.30±0.1 | 562.5 | 5.5 ± 0.7 | 3.0 ± 1.2 | 10.0 ± 3.8 | 0.7 ± 0.02 | 1.9 ± 0.2 | 91.8 ± 5.8 | 8.2 ± 5.8 |
| 2 | 1.94±0.1 | 760.5 | 2.2 ± 0.3 | - | - | - | - | - | - |
| 3 | 1.30±0.0 | 558.9 | - | - | - | - | - | - | - |
| 4 | 1.95±0.0 | 1271.8 | - | - | - | - | - | - | - |
| 5 | 1.64±0.0 | 1356.7 | 1.5 ± 0.2 | 2.4 ± 1.4 | 11.3 ± 4.7 | 1.7 ± 1.1 | 7.3 ± 4.3 | 37.0 ± 21.5 | 63.0 ± 21.5 |
| 6 | 1.78±0.0 | 1444.3 | - | - | - | - | - | - | - |
| 7 | 1.51±0.2 | 1451.9 | - | - | - | - | - | - | - |
| 8 | 1.23±0.1 | 812.5 | 1.1 ± 0.1 | 3.8 ± 0.4 | 19.4 ± 1.8 | 0.4 ± 0.06 | 2.6 ± 0.3 | 79.1± 14.8 | 20.9 ± 14.8 |
| 9 | 1.41±0.0 | 752.5 | - | - | - | - | - | - | - |
| 10 | 1.85±0.1 | 1209.0 | - | - | - | - | - | - | - |
| 11 | 2.19±0.0 | 1404.5 | 3.1 ± 0.3 | - | - | - | - | - | - |
| 12 | 2.03±0.1 | 1608.0 | - | - | - | - | - | - | - |
| 13 | 2.30±0.1 | 1382,3 | 5.7 ± 0.7 | 2.1 ± 0.8 | 9.4 ± 4.0 | 2.6 ± 0.7 | 9.8 ± 2.4 | 100.0 | 0.0 |
| 2 | 7.48±0.2 | 454.4 | 1.7 ± 0.1 | - | - | - | - | - | - |
| 3 | 7.93±0.0 | 579.0 | - | - | - | - | - | - | - |
| 4 | 6.18±0.0 | 684.3 | - | - | - | - | - | - | - |
| 5 | 5.88±0.1 | 661.1 | 1.3 ± 0.1 | 1.5 ± 0.8 | 10.4 ± 5.0 | 0.3 ± 0.1 | 2.3 ± 0.9 | 46.0 ± 20.3 | 54.0 ± 20.3 |
| 6 | 5.45±0.1 | 976.5 | - | - | - | - | - | - | - |
| 7 | 5.35±0.0 | 883.6 | - | - | - | - | - | - | - |
| 8 | 6.38±0.1 | 825.8 | 2.1 ± 0.1 | 4.1 ± 0.4 | 19.9 ± 2.4 | 1.7 ± 0.1 | 8.9 ± 0.8 | 94.1 ± 4.2 | 5.9 ± 4.2 |
| 9 | 6.42±0.2 | 619.8 | - | - | - | - | - | - | - |
| 10 | 7.58±0.2 | 984.2 | - | - | - | - | - | - | - |
| 11 | 6.64±0.0 | 1044.9 | 6.3 ± 0.2 | - | - | - | - | - | - |
| 12 | 7.51±0.1 | 1016.3 | - | - | - | - | - | - | - |
| 13 | 6.62±0.0 | 1334.3 | 6.3 ± 0.5 | 1.3±0.1 | 26.6±2.1 | 0.1±0.0 | 1.9±0.2 | 100±0.0 | 0.0 |
|  |  |  |  |  |  |  |  |  |  |
|  |  |  |  |  |  |  |  |  |  |
|  |  |  |  |  |  |  |  |  |  |

**Table 2SM (cont)**

| **Days** | **Temp** | ***p*CO_2_** | **BP** | **GZ** | **%BA_GZ_** | **BLV** | **%BA_BLV_** | **%LVP** | **%LysoVP** |
| --- | --- | --- | --- | --- | --- | --- | --- | --- | --- |
| 2 | 7.70±0.3 | 246.8 | 0.8 ± 0.06 | - | - | - | - | - | - |
| 3 | 7.63±0.1 | 175.4 | - | - | - | - | - | - | - |
| 4 | 6.11±0.2 | 156.1 | - | - | - | - | - | - | - |
| 5 | 6.06±0.0 | 134.9 | 1.7 ± 0.2 | 2.8 ± 1.7 | 15.0 ± 8.9 | 0.5 ± 0.0 | 2.5 ± 0.0 | 28.0 ± 19.8 | 72.0 ± 19.8 |
| 6 | 6.18±0.3 | 205.9 | - | - | - | - | - | - | - |
| 7 | 6.27±0.1 | 288.2 | - | - | - | - | - | - | - |
| 8 | 6.43±0.0 | 1458.9 | 2.6 ± 0.1 | 5.8 ± 2.4 | 21.6 ± 2.7 | 0.4 ± 0.06 | 1.4 ± 0.3 | 69.5± 21.6 | 13.0 ± 0.0 |
| 9 | 7.55±0.2 | 1553.2 | - | - | - | - | - | - | - |
| 10 | 6.90±0.1 | 1897.2 | - | - | - | - | - | - | - |
| 11 | 6.34±0.0 | 1759.2 | 4.0 ± 0.9 | - | - | - | - | - | - |
| 12 | 6.48±0.0 | 1803.4 | - | - | - | - | - | - | - |
| 13 | 6.39±0.0 | 2312.0 | 4.9 ± 0.5 | 2.5±0.4 | 23.5±1.6 | 0.2±0.1 | 2.0 ±1.3 | 6.04±0.0 | 90.07±3.47 |
| 2 | 10.64±0.1 | 503.6 | 1.7 ± 0.3 | - | - | - | - | - | - |
| 3 | 9.82±0.2 | 564.6 | - | - | - | - | - | - | - |
| 4 | 9.77±0.3 | 564.3 | - | - | - | - | - | - | - |
| 5 | 9.86±0.0 | 902.3 | 3.0 ± 0.3 | 0.07 ± 0.03 | 0.7 ± 0.2 | 1.7 ± 0.3 | 17.2 ± 4.0 | 74.6 ± 18.0 | 25.4 ± 18.0 |
| 6 | 10.49±0.1 | 864.8 | - | - | - | - | - | - | - |
| 7 | 11.24±0.0 | 996.7 | - | - | - | - | - | - | - |
| 8 | 10.19±0.0 | 939.9 | 7.5 ± 0.6 | 5.0 ± 0.05 | 27.0 ±8.7 | 1.2 ± 0.2 | 6.6 ± 0.3 | 62.0 ± 26.6 | 38.0 ± 26.6 |
| 9 | 10.26±0.1 | 446.2 | - | - | - | - | - | - | - |
| 10 | 10.87±0.0 | 988.9 | - | - | - | - | - | - | - |
| 11 | 10.07±0.0 | 1058.2 | 6.3 ± 1.0 | - | - | - | - | - | - |
| 12 | 9.83±0.0 | 1103.4 | - | - | - | - | - | - | - |
| 13 | 10.27±0.1 | 1359.3 | 9.0 ± 1.1 | 4.8 ± 1.1 | 53.1 ± 6.2 | 1.1 ± 0.4 | 13.3 ± 2.5 | 100.0 | 0.0 |
| 2 | 10.43±0.2 | 259.3 | 1.0 ±0.4 | - | - | - | - | - | - |
| 3 | 9.83±0.0 | 168.5 | - | - | - | - | - | - | - |
| 4 | 9.69±0.1 | 187.5 | - | - | - | - | - | - | - |
| 5 | 9.83±0.0 | 186.7 | 2.4 ± 0.4 | 1.7 ± 0.6 | 12.8 ± 4.9 | 0.2 ± 0.07 | 1.5 ± 0.6 | 79.6 ± 14.4 | 20.4 ± 14.43 |
| 6 | 10.59±0.1 | 155.3 | - | - | - | - | - | - | - |
| 7 | 11.33±0.0 | 1101.3 | - | - | - | - | - | - | - |
| 8 | 10.25±0.1 | 1243.3 | 6.6 ± 2.4 | 3.5 ± 0.6 | 18.5 ± 1.1 | 2.6 ± 0.4 | 13.1 ± 2.1 | 63.0 ± 12.1 | 37.0 ± 12.1 |
| 9 | 10.21±0.0 | 1457.8 | - | - | - | - | - | - | - |
| 10 | 11.00±0.3 | 1969.6 | - | - | - | - | - | - | - |
| 11 | 9.98±0.0 | 1730.7 | 4.6 ± 0.5 | - | - | - | - | - | - |
| 12 | 9.83±0.1 | 1595.2 | - | - | - | - | - | - | - |
| 13 | 10.37±0.1 | 2318.3 | 13.0 ± 1.5 | 0.9 ± 0.3 | 22.2 ± 7.8 | 0.2 ± 0.04 | 5.3 ± 0.6 | 43.8 ± 11.1 | 58.5 ± 11.1 |
