# Supplementary figures and images for: Warming and CO2 Enhance Arctic Heterotrophic Microbial Activity
Source: Front Microbiol. 2019 Mar 20;10:494. doi: 10.3389/fmicb.2019.00494 (PMC6436474; doi:10.3389/fmicb.2019.00494)

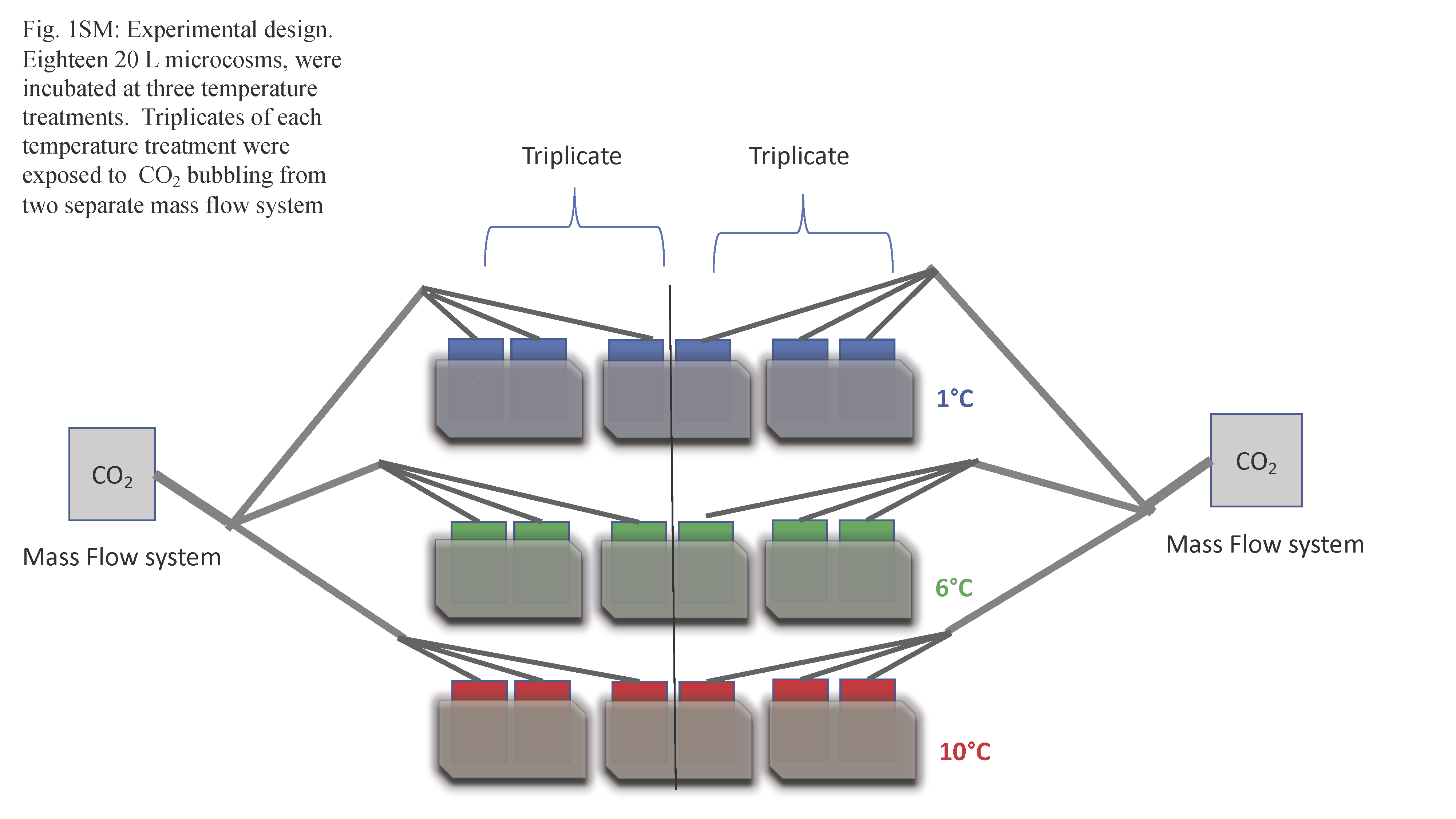

Supplement: Supplementary file 2 [file Image_1.TIF]

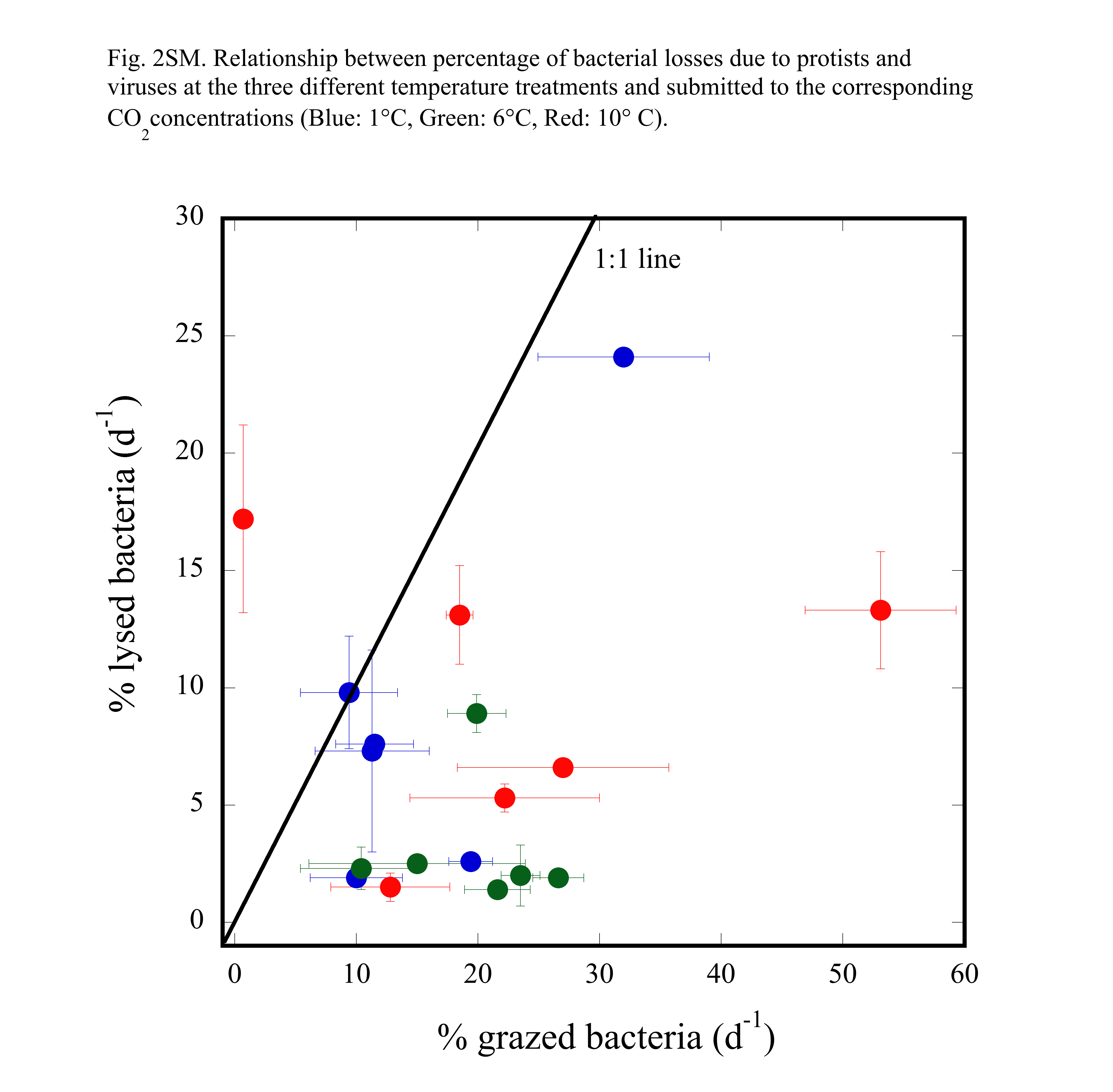

Supplement: Supplementary file 3 [file Image_2.tif]
